# A reexamination on how behavioral interventions can promote household action to limit climate change

**DOI:** 10.1038/s41467-020-14653-x

**Published:** 2020-02-14

**Authors:** Paul C. Stern

**Affiliations:** Social and Environmental Research Institute, Greenfield, MA USA

**Keywords:** Climate-change mitigation, Psychology and behaviour

**Arising from** Claudia F. Nisa et al. *Nature Communications* 10.1038/s41467-019-12457-2

There has been a long-standing debate on whether and how behavioral interventions can reduce the contribution of household actions to climate change. Nisa et al.^1^ review randomized field trials and find that behavioral interventions, taken alone, have very little effect on household actions. Although many readers might inappropriately conclude that such interventions have no meaningful place in efforts to limit climate change, a long-standing body of multimethod research shows that when combined with non-behavioral interventions to address context-specific barriers to change, behavioral interventions can greatly increase the impact of household actions. Further research should focus on high-impact actions, use multiple research methods, and combine different types of interventions to explore how and under what conditions behavioral interventions can have practical value.

Behavioral interventions are roughly defined in the literature to include interventions that involve neither command-and-control regulations nor financial incentives—e.g., information provision, appeals to values and norms, engagement, and restructuring choice options (so-called nudges). Despite the strong analysis in ref. ^1^, an inference of no practical value is inappropriate for three main reasons. First, the analysis covers only behavioral interventions acting alone. However, as elaborated below, long-standing literature indicates that the most effective ways to use behavioral interventions to change household actions affecting GHG emissions are not alone, but in combination with financial and other interventions^2–4^. The findings in ref. ^1^ will not surprise readers familiar with that literature. But it contributes by showing through careful methodological analysis that behavioral interventions on their own have small average effects on the household behaviors studied, even when the effects are statistically significant. These findings may help stimulate behavioral researchers to rethink how best to use behavioral interventions for real-world impact.

Second, the paper does not distinguish clearly between frequently occurring behaviors (e.g., travel to work, adjusting room temperatures, changing diet), whose effects must be aggregated through multiple repetitions, and infrequent behaviors, usually involving investments, whose effects are almost fully achieved by a single action (e.g., upgrading the energy efficiency of building shells, adopting more energy-efficient home power systems and vehicles). All but four of the randomized controlled trials reviewed examine the first class of behaviors (the others concern appliance choices), but the authors do not fully consider the implications of that fact. The paper barely addresses the household behaviors with the greatest potential for reducing GHG emissions on a time scale of years to decades, which are of the single-action type^3^. These actions include home weatherization and acquisition of fuel-efficient vehicles, which both have a far greater technical potential for emissions reduction than appliance purchases^3^. Other one-time actions that have become more widespread in recent years, such as adoption of photovoltaic energy systems and electric vehicles that can run on them, arguably also have much greater potential importance than appliance purchases or frequently occurring behaviors. There has long been evidence that the determinants for the two types of action are different and that intrapersonal factors have less effect on investments^5^. Because this paper barely addresses such high-impact behaviors, the practical relevance of the meta-analysis is open to question. For example, the problem of establishing habits, discussed in ref. ^1^ as whether the behaviors stand the test of time, has an important place in that paper, but seems irrelevant for one-time behaviors.

The meta-analysis sample reflects the history of behavioral researchers’ focus on frequently repeated behaviors^4,6^ and may reflect the difficulty of organizing randomized control studies of higher-impact one-time household investments. This gap in the literature is important: a focus on potentially high-impact behaviors is critical for behavioral research to make its greatest contribution, especially as the determinants of the two kinds of behavior may well be different^4,5^. Research on high-impact behaviors uses various non-experimental methods, is limited and fragmentary, but yet suggests some conclusions of practical value^4,7–9^. Because a review restricted to randomized clinical trials omits the highest-impact household actions, its conclusions need to be qualified carefully before drawing general inferences about the potential impact of behavioral interventions. A focus on high-impact actions points to the great importance of context-specific barriers to change and the value of combining behavioral with regulatory and financial interventions.

Third, the paper acknowledges, but is unable to explore, the potential for behavioral interventions to have practical impact when combined with other intervention types. It seems implicitly to assume that for each type of behavior there is a single, universal best estimate of the amount of change that can be achieved by each type of intervention. For interactions of intervention types, it seems to presume that there is a single best estimate of each interaction effect that could be made by analyzing appropriately collected data. The evidence from multiple research methods indicates otherwise^2,4,9–11^. It indicates that the effects of behavioral interventions depend on multiple contextual factors, which may be physical, institutional, economic, cognitive, attitudinal, etc., and which can create barriers to change. It shows that the greatest behavioral plasticity is achieved by intervention packages that adequately address the specific barriers to change that apply to target behaviors, populations, and contexts. In short, behavioral interventions can have their greatest impact when other interventions reduce non-behavioral barriers. For example, behavioral response to identical financial incentives for home weatherization has varied tenfold due to non-technical and non-economic factors in implementation^12^.

Multi-method research has generated design principles for practical interventions that integrate behavioral and other intervention types, particularly for high-impact actions, and that can be adapted to a variety of contexts^2,4,13,14^. The principles emphasize the needs for marketing to get households to consider taking high-impact actions, providing valid information from credible sources at points of decision, making information about choices simple, and providing credible quality assurance for household investments in low-emissions technologies^4,7,13^. Behavior- and context-specific interventions may seem less attractive than economy-wide interventions such as carbon taxes and markets, but because limiting climate change is an urgent priority and because these approaches face major challenges of adoption and implementation^15^, focusing on high-impact behaviors is important now and will remain so as economy-wide interventions are developed.

The idea that responsiveness to behavioral interventions is context specific, and not a constant, is not readily tested by controlled experimentation, but it has robust support. Additionally, it suggests a fundamental limitation of treating controlled experimentation as the gold-standard methodology in this domain. What can be learned from randomized controlled trials can be valuable, but its limitations for drawing broad conclusions for policy need to be taken seriously.

Analysis of the findings of ref. ^1^ reveals the limits of generalizing from single variable analyses of behavioral interventions, focusing on frequently repeated actions, and seeking universal, context-independent, generalizations about the effects of particular intervention strategies on particular types of behavior. As other research strongly indicates, well targeted behavioral interventions, particularly focused on infrequent, higher-impact actions, can have significant impact when non-behavioral barriers are reduced. Useful practical advice can come from research focused on high-impact actions, using multiple research methods, considering contextual effects, and examining promising combinations of behavioral and other intervention types, to address and overcome barriers to change for specific behaviors in specific contexts^3,4,7^.

## Data Availability

Data sharing not applicable.
